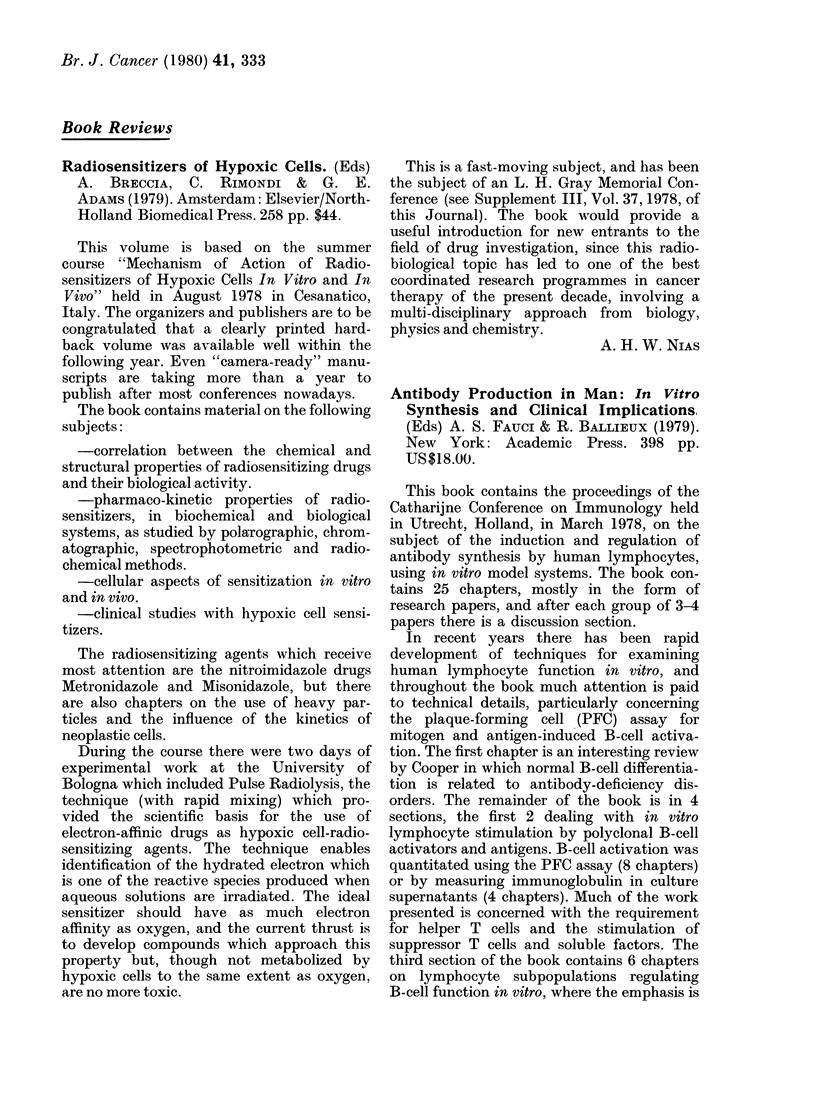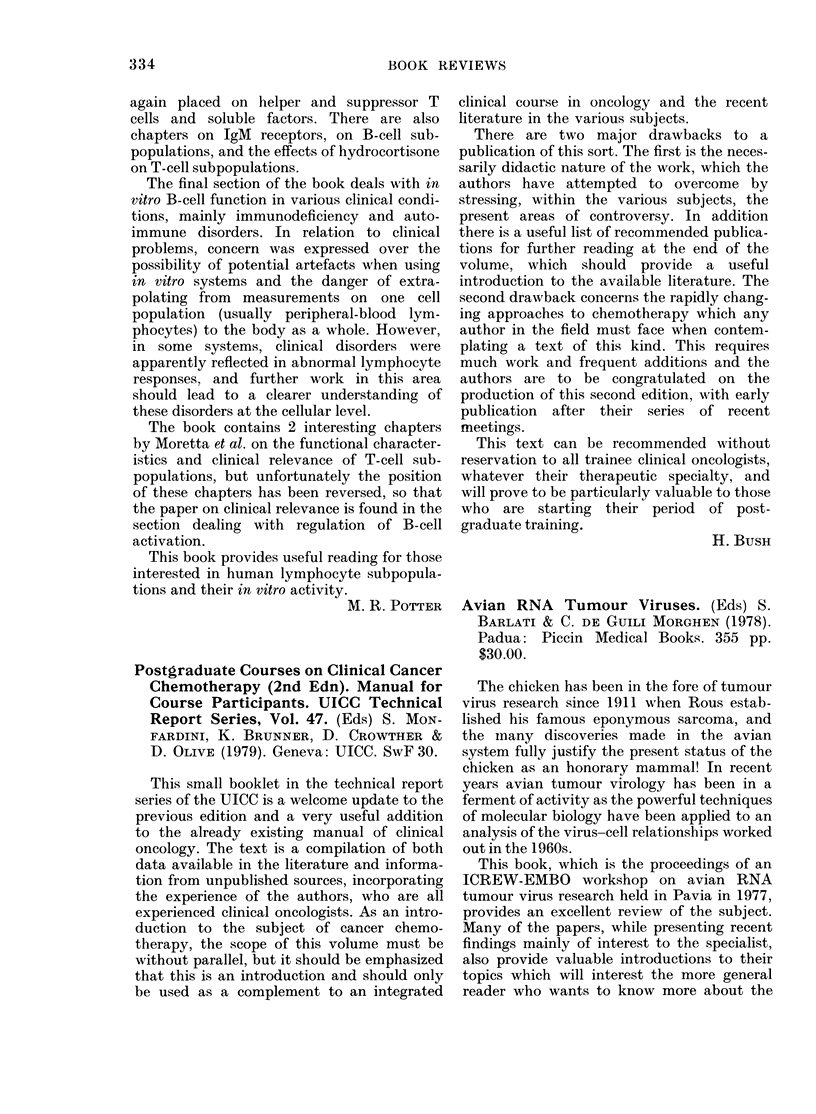# Antibody Production in Man: In Vitro Synthesis and Clinical Implications

**Published:** 1980-02

**Authors:** M. R. Potter


**Antibody Production in Man: *In Vitro* Synthesis and Clinical Implications.** (Eds) A. S. Fauci & R. Ballieux (1979). New York: Academic Press. 398 pp. US$18.00.

This book contains the proceedings of the Catharijne Conference on Immunology held in Utrecht, Holland, in March 1978, on the subject of the induction and regulation of antibody synthesis by human lymphocytes, using *in vitro* model systems. The book contains 25 chapters, mostly in the form of research papers, and after each group of 3–4 papers there is a discussion section.

In recent years there has been rapid development of techniques for examining human lymphocyte function *in vitro*, and throughout the book much attention is paid to technical details, particularly concerning the plaque-forming cell (PFC) assay for mitogen and antigen-induced B-cell activation. The first chapter is an interesting review by Cooper in which normal B-cell differentiation is related to antibody-deficiency disorders. The remainder of the book is in 4 sections, the first 2 dealing with *in vitro* lymphocyte stimulation by polyclonal B-cell activators and antigens. B-cell activation was quantitated using the PFC assay (8 chapters) or by measuring immunoglobulin in culture supernatants (4 chapters). Much of the work presented is concerned with the requirement for helper T cells and the stimulation of suppressor T cells and soluble factors. The third section of the book contains 6 chapters on lymphocyte subpopulations regulating B-cell function *in vitro*, where the emphasis is again placed on helper and suppressor T cells and soluble factors. There are also chapters on IgM receptors, on B-cell subpopulations, and the effects of hydrocortisone on T-cell subpopulations.

The final section of the book deals with *in vitro* B-cell function in various clinical conditions, mainly immunodeficiency and autoimmune disorders. In relation to clinical problems, concern was expressed over the possibility of potential artefacts when using *in vitro* systems and the danger of extrapolating from measurements on one cell population (usually peripheral-blood lymphocytes) to the body as a whole. However, in some systems, clinical disorders were apparently reflected in abnormal lymphocyte responses, and further work in this area should lead to a clearer understanding of these disorders at the cellular level.

The book contains 2 interesting chapters by Moretta *et al.* on the functional characteristics and clinical relevance of T-cell subpopulations, but unfortunately the position of these chapters has been reversed, so that the paper on clinical relevance is found in the section dealing with regulation of B-cell activation.

This book provides useful reading for those interested in human lymphocyte subpopulations and their *in vitro* activity.

M. R. POTTER